# Beaver dam capacity of Canada’s boreal plain in response to environmental change

**DOI:** 10.1038/s41598-020-73095-z

**Published:** 2020-10-08

**Authors:** Nichole-Lynn Stoll, Cherie J. Westbrook

**Affiliations:** Department of Geography and Planning, University of Saskatchewan, Saskatoon, SK S7N 5C8 Canada

**Keywords:** Hydrology, Climate-change impacts, Ecological modelling, Boreal ecology, Riparian ecology

## Abstract

Environmental changes are altering the water cycle of Canada’s boreal plain. Beaver dams are well known for increasing water storage and slowing flow through stream networks. For these reasons beavers are increasingly being included in climate change adaptation strategies. But, little work focuses on how environmental changes will affect dam building capacity along stream networks. Here we estimate the capacity of the stream network in Riding Mountain National Park, Manitoba, Canada to support beaver dams under changing environmental conditions using a modelling approach. We show that at capacity, the park’s stream network can support 24,690 beaver dams and hold between 8.2 and 12.8 million m^3^ of water in beaver ponds. Between 1991 and 2016 the park’s vegetation composition shifted to less preferred beaver forage, which led to a 13% decrease in maximum dam capacity. We also found that dam capacity is sensitive to the size of regularly-occurring floods—doubling the 2-year flood reduces the park’s dam capacity by 21%. The results show that the potential for beaver to offset some expected climatic-induced changes to the boreal water cycle is more complex than previously thought, as there is a feedback wherein dam capacity can be reduced by changing environmental conditions.

## Introduction

Canada’s boreal forest has long been important beaver, *Castor canadensis*, habitat^1^. Intensive trapping of beaver in the 1700s through the 1800s caused near extirpation of the species in the boreal forest^2^. Population recovery began in the 1930s via re-introduction and conservation programs^3^. Beaver are keystone in supporting food security of Indigenous people residing in the boreal forest through providing a source of meat and being ecologically influential^4,5^. They are viewed as an ecologically influential species as they profoundly alter the aquatic ecosystems they occupy^2^ with benefits for freshwater biodiversity^6^ and terrestrial wildlife^7^. Beaver alter aquatic ecosystems primarily through engineering activities—dam and canal building—which modifies ecosystem-forming processes^8^. For example, beaver dams raise and stabilize water tables^9,10^, alter stream hydrographs^11,12^, enhance channel and riparian area sediment retention^13,14^ and create hydrologically complex, multi-channel networks^9,15^. The suite of changes beaver dams make to aquatic ecosystems creates a multitude of desired ecological feedbacks that collectively enhance ecosystem resilience to disturbance^16^.

Environmental changes resulting from the cumulative impacts of disturbance-recovery dynamics and climate change^17^ are expected to considerably alter the ecological and hydrological character of the boreal forest over time^18^. Beaver are likely an important part of enhancing the resiliency of the boreal forest to environmental change^19,20^. Yet, recent and continued environmental changes will also impact the beaver population. Increases in the occurrence and severity of wildfires, for example, are expected in the future which will change the vegetation composition of boreal forests^21^. Fires renew beaver food sources, especially willow and aspen which they prefer, providing quality habitat for beaver 5–30 years afterwards^22–25^. Further, the growing oil and gas extraction industry in the western portion of Canada’s boreal forest^26^ is causing loss of beaver habitat but creation of new habitat in engineered landforms^27^. The way in which the beaver population will respond to the set of ongoing environmental changes is likely complex. For example, beavers may respond to climate change by modestly expanding their range, especially northward^28^, and densifying in their current range^29^.

**Figure 1:**
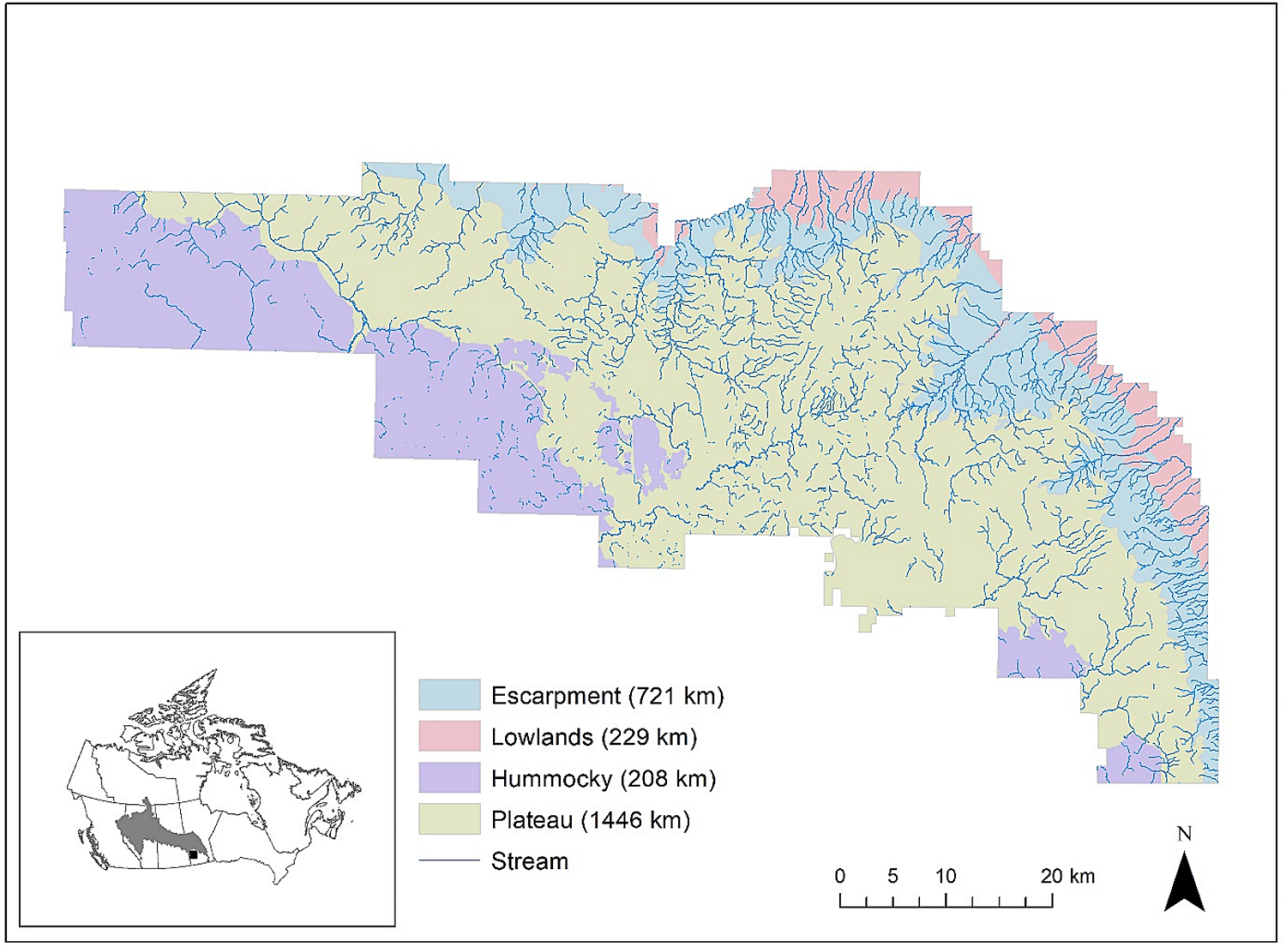
The study site, Riding Mountain National Park. Depicted in the park is the stream drainage network occurring within each of the four main physiographies (main image). The inset situates the park (black square) within Canada’s Boreal Plains ecozone (shaded).

Many studies have compared beaver site occupancy with habitat characteristics; recent examples include Smeraldo et al.^30^ and Scrafford et al.^31^. But, few studies have asked how changing beaver habitat characteristics owing to environmental change will affect how many and where beaver dams are built along stream networks, information critical to understanding the effects of climate change on water resources. In this study we assess the landscape capacity of the boreal forest to support beaver dams under changing environmental conditions. We use the example of Riding Mountain National Park in Canada (Fig. 1) because the park is located at the southeastern limit of the boreal plain which is most vulnerable to climate change^18^; the park also has long-term records of beaver caches. Beaver were extirpated from the park and adjacent areas via trapping before 1930 and reintroduced in 1947^32^. Ongoing aerial surveys of food caches since 1973 are used by the park to track beaver population dynamics^33^. To achieve our aim, we ran the BRAT model^34^ (see “Methods”) across Riding Mountain National Park under various scenarios to assess: (1) the capacity of the park’s 2604 km of streams to support beaver dams; (2) how changes in forest composition over the past three decades have affected beaver dam capacity; and (3) how climatic-induced changes in streamflow will influence beaver dam capacity. We find that the park can support a large number of beaver dams, and that beaver ponds store a considerable volume of water. However, changes to park vegetation cover and the size of regularly occurring floods will reduce the number of beaver dams the park can support. The approach used should hold significant value for use elsewhere, not only for understanding changes in beaver habitat and dam capacity under previously recorded change, but also for estimating changes under future land use and land cover change scenarios.

## Results

### Beaver dam capacity and pond water storage

**Figure 2:**
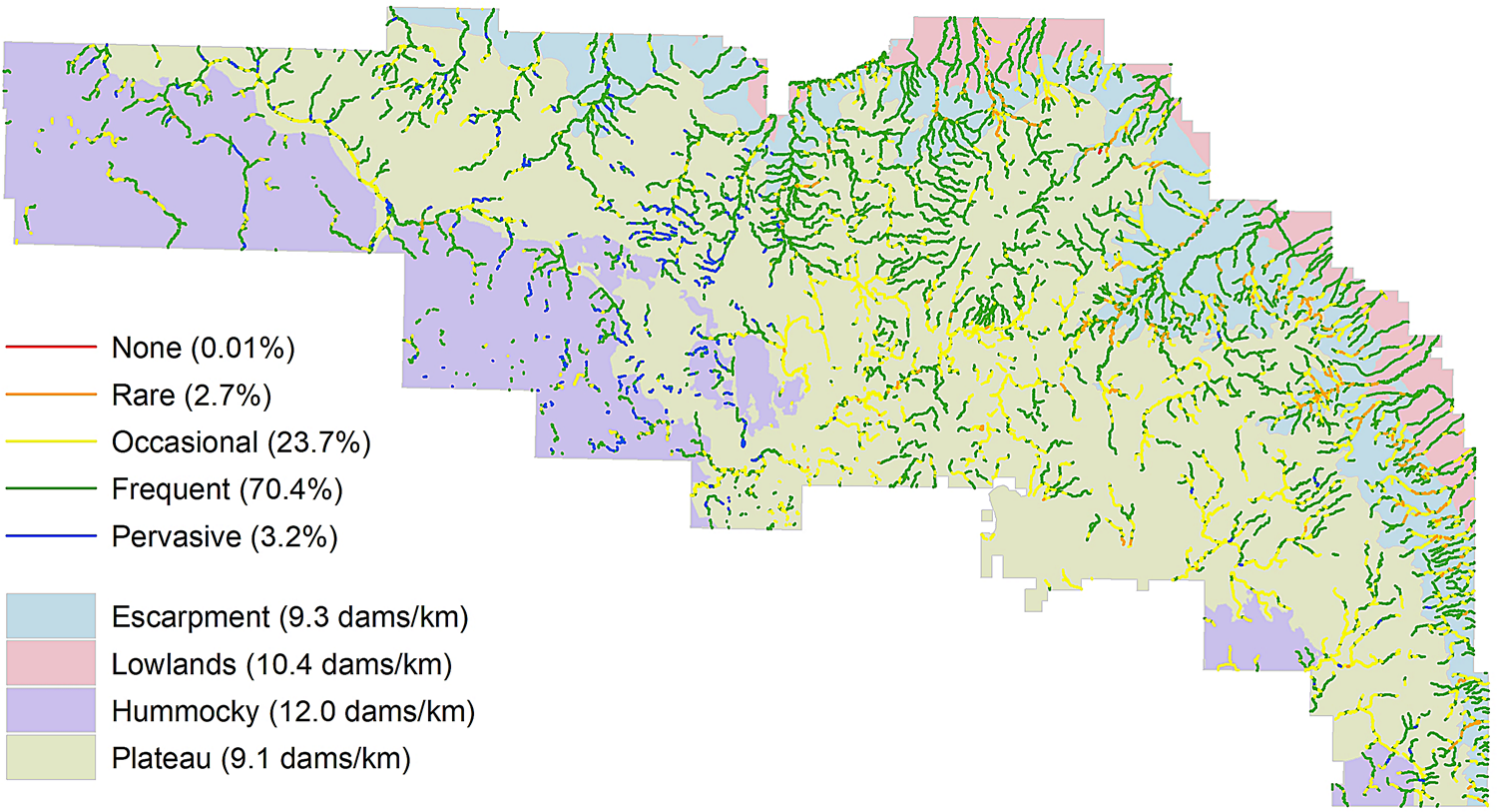
Modelled beaver dam capacity (by capacity category) at the reach scale (300 m segment) for the stream network within Riding Mountain National Park. Maximum beaver dam density by physiography is reported.

The modelling results indicate Riding Mountain National Park can support a large number of beaver dams. We estimate the park has a dam capacity of 24,690 dams or 9.5 dams per stream km based on 2016 imagery (Fig. 2). At 70% of the stream network (1833 km), the *frequent* dam density category is the largest. Little of the stream network (3%) was in the *rare* and *none* dam density categories. Using the park modelled dam capacity estimate, a bootstrapped median beaver pond size of 1419 m^2^ (95% confidence interval, CI, of [1157, 1704 m^2^]) and the water storage algorithm of Karran et al.^35^ (see “Methods”), we estimate the park has a beaver pond storage capacity 10.4 million m^3^ of water (CI of [8.2, 12.8 million m^3^]).

Modelled dam density varies across the park. Dam capacity is greatest in the plateau (13,105 dams) and lowest in lowlands (2389). The main cause of this difference is the length of the stream network. When dam capacity is scaled to stream length, there is less difference among physiographies. The upper limit of dam density is highest (12.0 dams per stream km) in the hummocky physiography and lowest in the plateau and escarpment physiographies (~ 9.2 dams per stream km in each).

**Figure 3:**
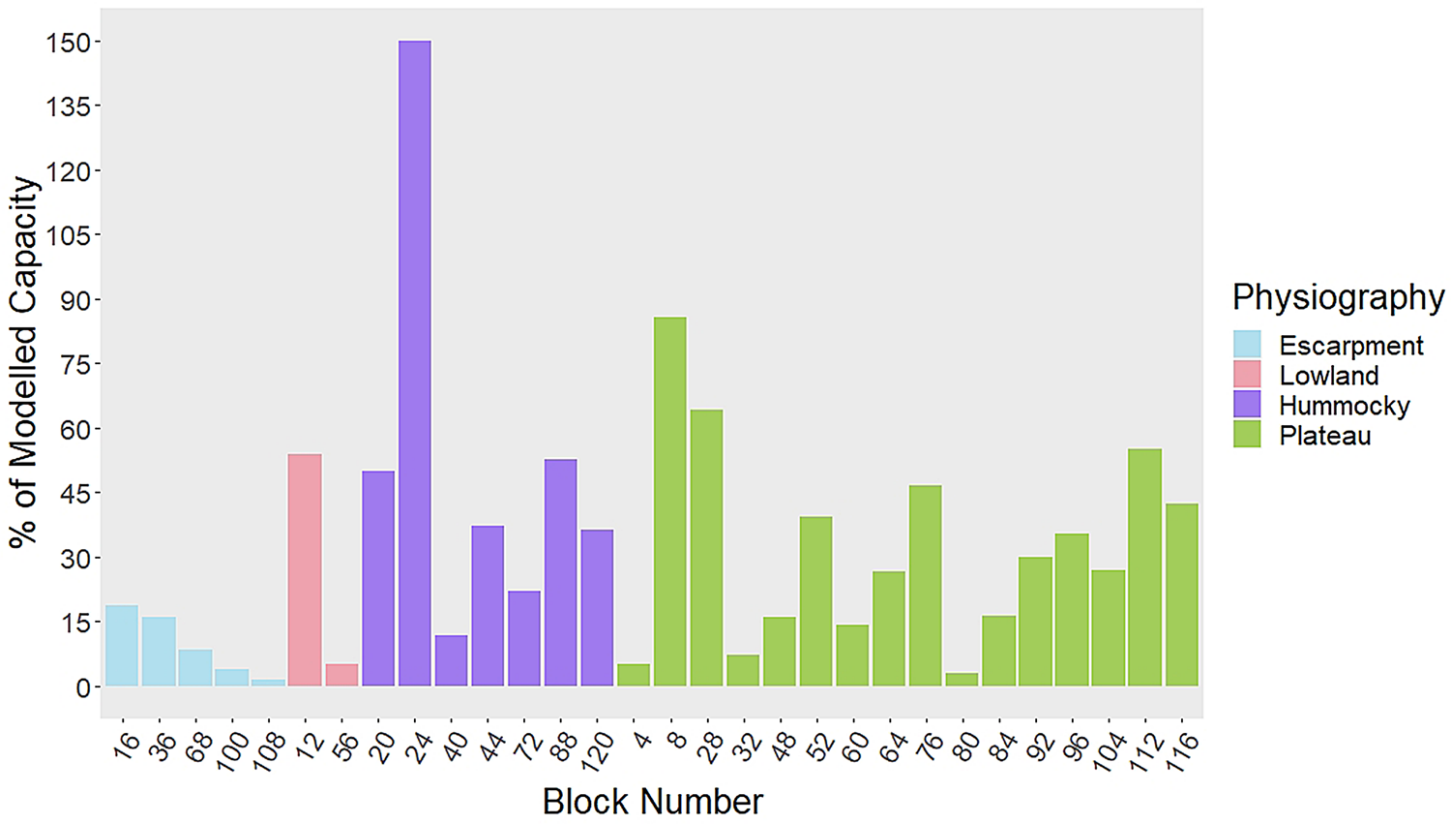
Beaver dams, determined from observations made in the long-term survey blocks across the park (see S1), are present at approximately 22% of capacity based on 2004 high-resolution imagery the 2003 BRAT results. Average proportion of dams in relation to modelled capacity for each physiography is: 10% escarpment, 51% hummocky, 30% lowlands, and 32% plateau.

**Figure 4:**
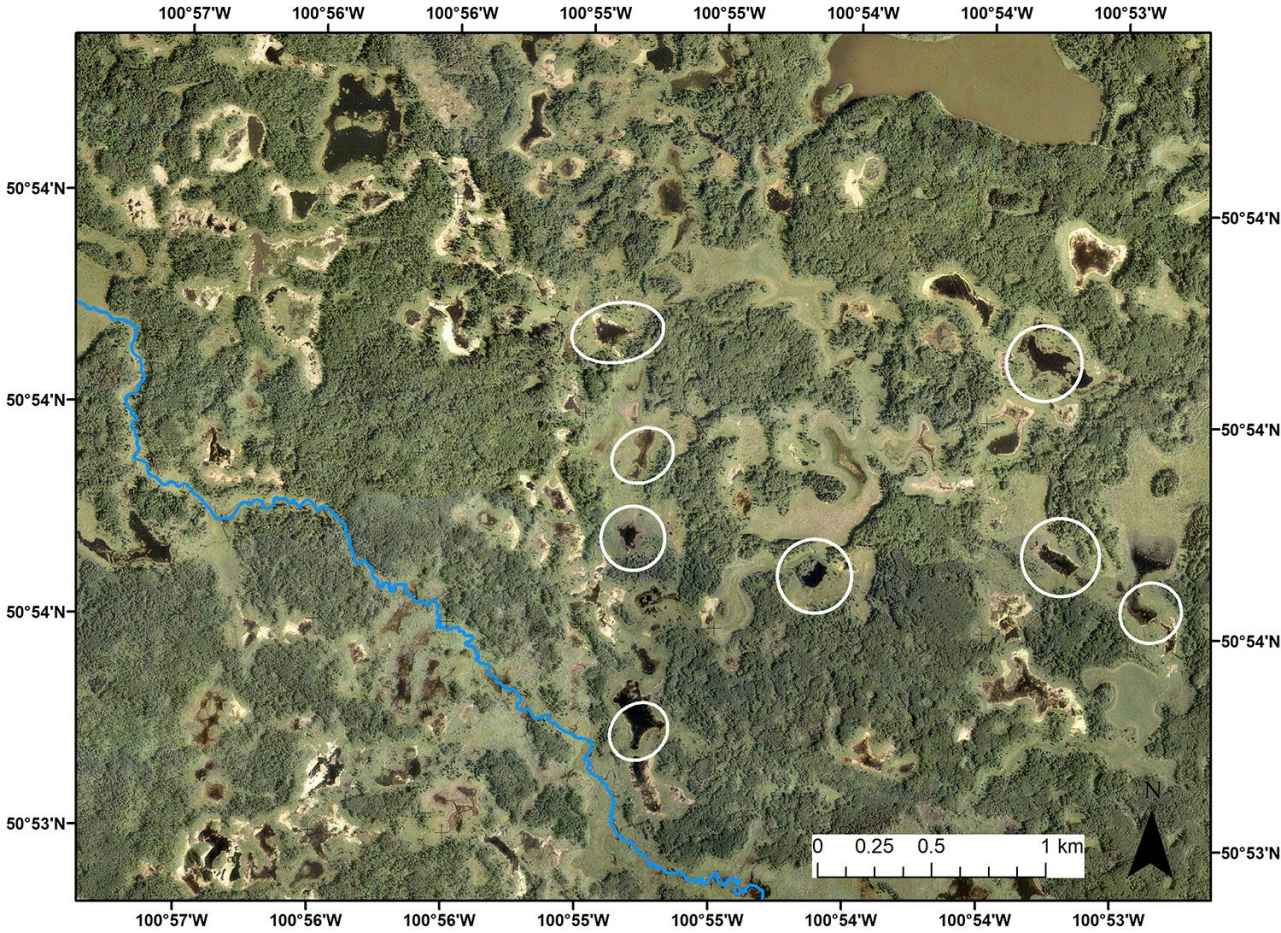
Aerial photograph (0.63 m resolution) from 2004 showing examples of beaver dams (circled) occurring in off-channel locations. The air photo is owned by Riding Mountain National Park and is used with permission. The blue line depicts the stream channel, the shapefile for which was sourced from the publicly available Manitoba Land Initiatives archive, https://mli2.gov.mb.ca/water_resources/index.html. The map was generated using ESRI ArcMap 10.5.

Beaver dams, determined from observations made in survey blocks scattered across the park (120 km^2^; see “Methods”), are present at approximately 22% of capacity (Fig. 3). There was, however, high variation in dam capacity by physiography. Beaver dams in the escarpment occur at the lowest percentage of capacity (10%) and in the hummocky at the highest percentage of capacity (51%). Dams occur in the lowland and plateau at 30% and 32% of capacity, respectively. One survey block (no. 24) had more beaver dams than the modelled capacity (150%)—this survey block was in the hummocky physiography. The BRAT model used to estimate beaver dam capacity only considers dams occurring along the river network. However, in conducting the analysis of beaver dam counts in the survey blocks, we observed a number of beaver dams in off-channel locations (Fig. 4), especially in the hummocky physiography and along the hummocky-plateau boundary.

### Impacts of changing vegetation composition on beaver dam capacity

**Figure 5:**
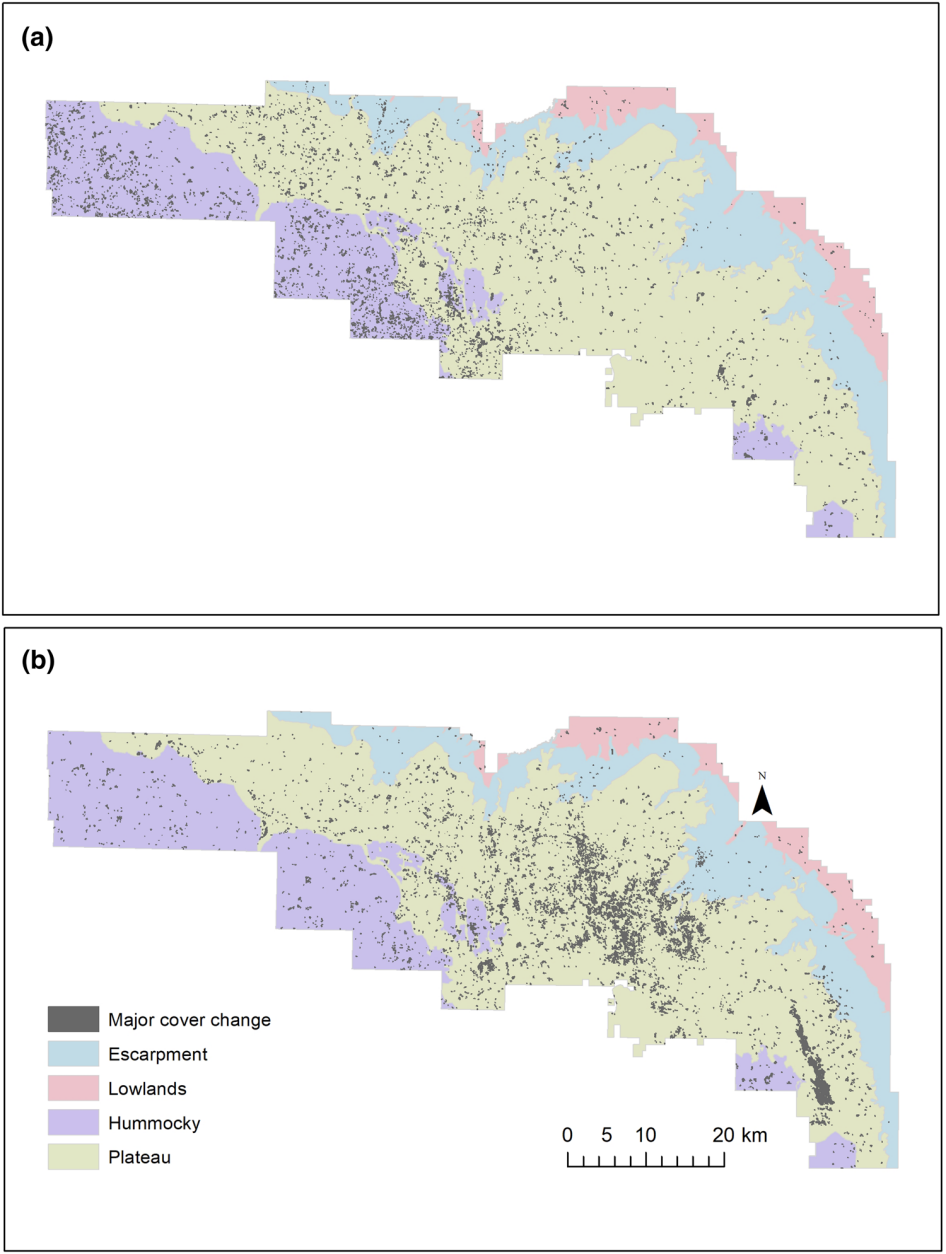
Places where major land cover change was detected in Riding Mountain National Park between 1991 and 2003 (**a**), and 2003 and 2016 (**b**).

**Figure 6:**
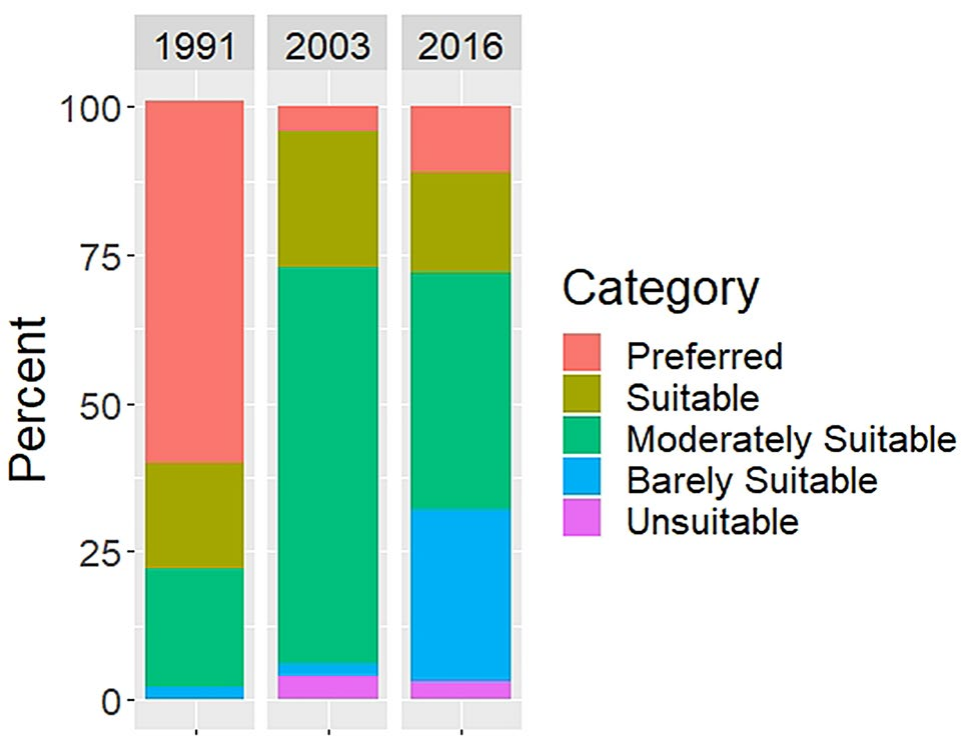
Suitability of vegetation cover for beaver foraging in places identified as changed between 1991 and 2016 on Fig. 5.

Vegetation cover of Riding Mountain National Park has changed over the last 25 years (Fig. 5). Overall changes were small—there was a 2.8% change in vegetation cover from 1991 to 2003, and an additional 3.4% change from 2003 to 2016. Much of the change between 1991 and 2003 occurred in the western part of the park in the hummocky and plateau physiographies while changes from 2003 to 2016 primarily occurred in the central and southeastern areas of the park in the plateau physiography. The largest vegetation change from 1991 to 2003 was a 23.2 km^2^ increase in marsh. The major vegetation change from 2003 to 2016 was a 28.2 km^2^ decrease in mixedwood. In places where vegetation cover change occurred, preferred beaver forage was reduced and replaced with less suitable beaver forage (Fig. 6).

**Figure 7:**
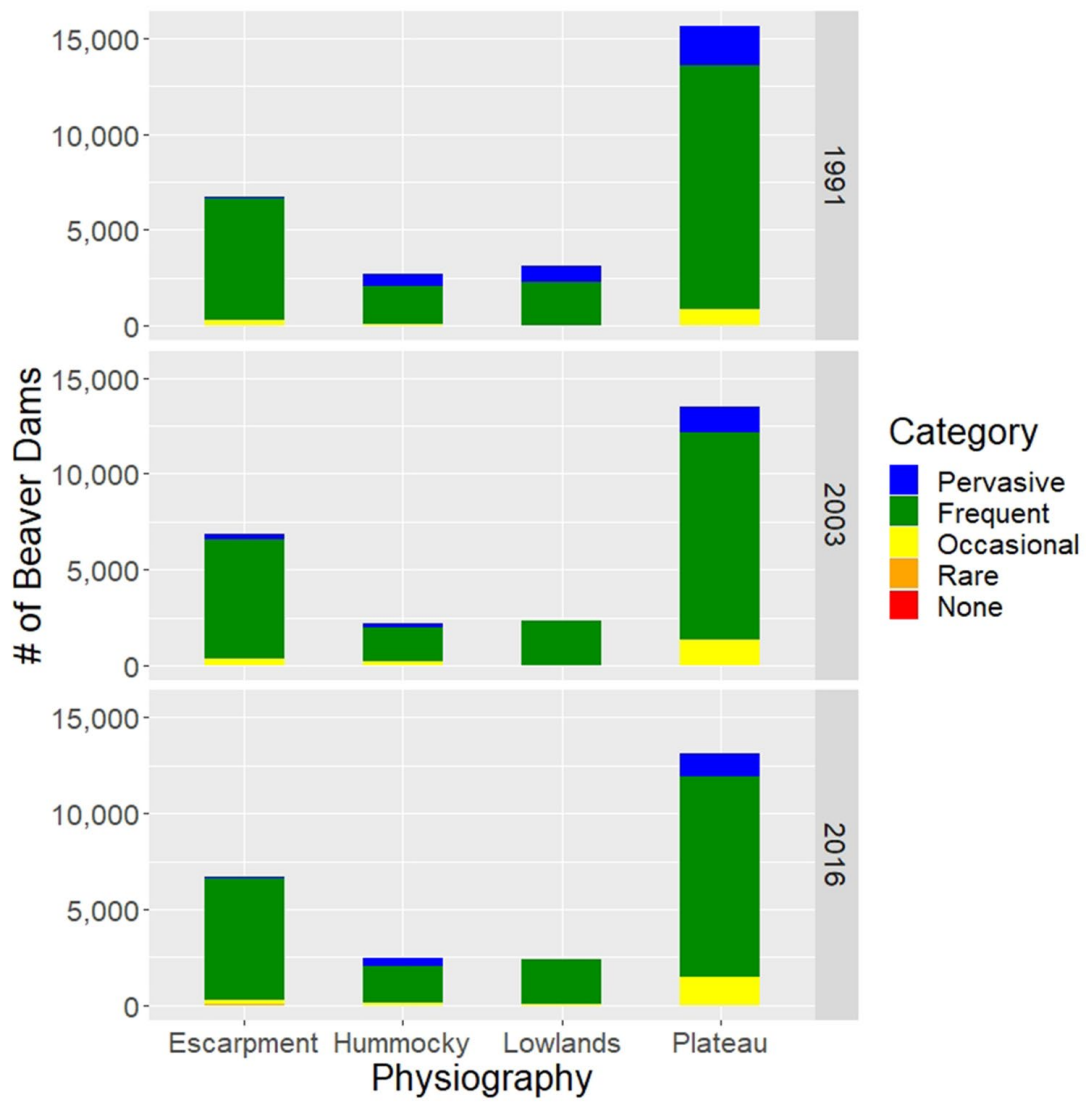
Comparison of beaver dam capacity by physiography as modelled using the vegetation cover in 1991, 2003 and 2016.

Beaver can only build dams in habitats where there is suitable and sustainable forage^34,36^ and so we estimated beaver dam capacity for the park by running the BRAT model with the 1991, 2003 and 2016 vegetation composition. The model results indicate streams in Riding Mountain National Park could support 14% more beaver dams in 1991 than in 2003 or 2016 (Fig. 7). The largest changes from 1991 and 2003 occurred within the *pervasive* and *occasional* dam density categories. Specifically, there was three times greater stream length in the pervasive dam density category in 1991 than in 2003 or 2016, which occurred primarily in the southern part of the park. Further, there was a third less stream length in the *occasional* dam density category in 1991 than in 2003 or 2016. Correspondingly, the estimated upper limit of water storage capacity in beaver ponds lowered from 12.0 million m^3^ in 1991 to 10.4 million m^3^ in 2003, a reduction of 13.3%. Beaver dam capacity and beaver pond water storage capacity in the park were similar when the model was run with 2003 vs 2016 vegetation cover (median ~ 10.4 million m^3^ of water for each year).

### Impacts of stream flow changes on beaver dam capacity

**Figure 8:**
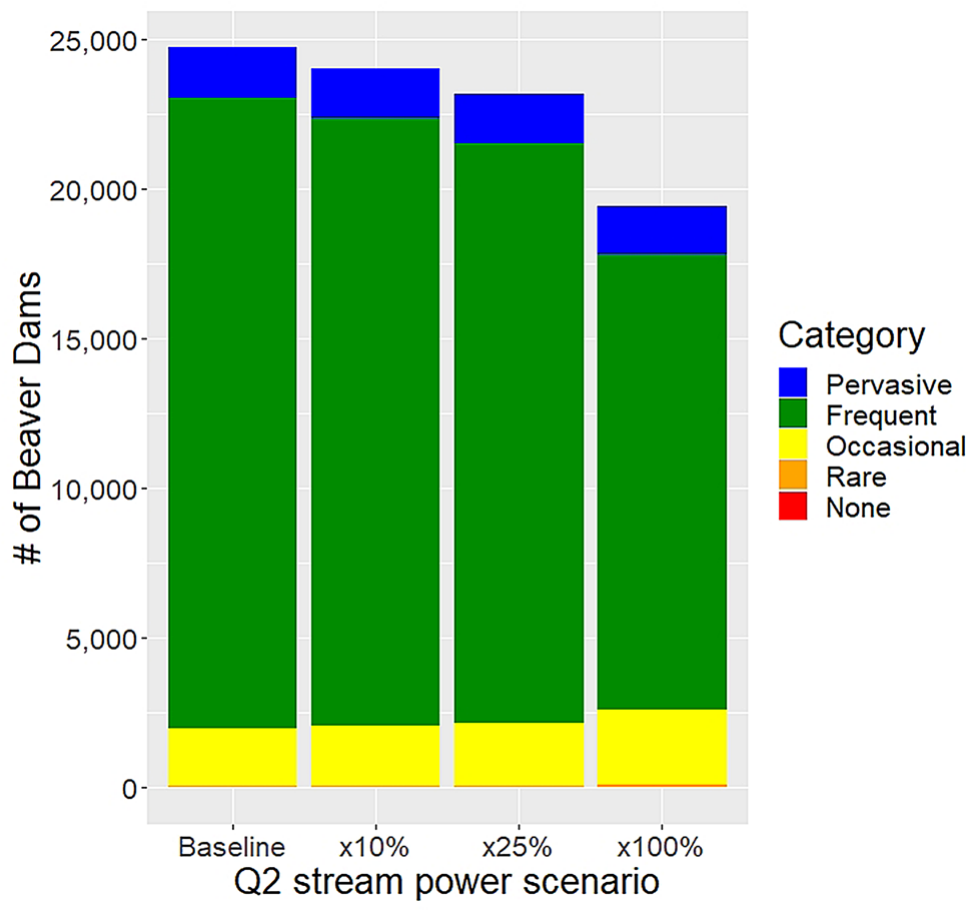
Model estimates of number of beaver dams in each dam density category for the stream flow change scenario analysis.

In addition to suitable forage, beaver require a reliable water source in order to build dams. On streams, what is suitable is perennial flow without large floods happening too often such that dams regularly fail^37^. The BRAT model uses the stream power associated with the two-year recurrence interval peak flood (Q2) to infer the likelihood that a beaver dam will persist once built^34^. Knowing that climate changes are already leading to wetter conditions in this part of Canada^38^ and are likely to continue into the future^19^, we ran scenarios in the BRAT model across the park wherein only Q2 was modified to assess how climatic-induced changes to streamflow might influence beaver dam capacity (see “Methods”). We found that smaller increases in the magnitude of Q2 modestly reduces the capacity of the park’s stream network to support beaver dams (Fig. 8). Increasing Q2 by 100% though reduces beaver dam capacity in the park by 21%. The decreases in dam capacity were most noticeable in the *frequent* dam density category, which decreased from comprising 70% of the stream network to 52% of it with a doubling of Q2. The stream segments originally classified in the *frequent* dam density category under Q2 shifted to being in the *occasional* (increase of 13%) and *rare* dam density categories (increase of 8%) in the 100% Q2 scenario.

## Discussion

The model predictions of beaver dam capacity presented here have at least three important implications. First, Riding Mountain National Park can support a large number of beaver dams, although dam capacity varies across the park. The many ecological and hydrological benefits provided by beaver dams are closely related to their frequency along a stream course^39^. While high, an average of nine dams per stream km density is not unrealistic for this region. Historic (pre fur trade) beaver occupation was at least this high^40^, and there are reports of current dam density exceeding 10 per km of stream in the literature^11,41^. High dam density is possible in Riding Mountain National Park, like much of the boreal forest in Canada, because it has an abundance of beaver-preferred vegetation. Beaver prefer aspen, poplar and cottonwood trees and willow, using these species for food and construction materials^42–44^. Mixedwood stands—comprised of aspen and poplar and an understory of willow and beaked hazelnut^45^—are extensive in the park. If the park reached its dam capacity, we estimate beaver dams would impound between 8.2 and 12.8 million m^3^ of water. To put that in perspective, the province of Manitoba is well known for hydroelectric dams. One of the largest hydroelectric dams in the province is the Limestone; it has a water storage capacity of just under 3 million m^3^^46^. Thus there is potential for impoundment of large volumes of water in beaver ponds with considerable impact to the park’s streamflow regime. Beaver-mediated enhanced water storage in the boreal plain is likely to become increasingly important with climate change as shallow ponds dry out^20,47^.

The distribution of beaver dams across the park is spatially variable. Our study area has different physiographies that differ in their topography and geology. Although all four physiographies have suitable beaver habitat, model runs indicate maximum beaver dam density in the escarpment and plateau physiographies is lower than in the hummocky and lowland physiographies. The differences in dam density results from the combined influence of availability of preferred vegetation adjacent to the stream network composition and the steepness of stream channels^22^. Where beaver-preferred vegetation is widely available near streams, flatter landscapes tend to support higher dam densities^48^. The hummocky and lowland physiographies are both low gradient environments. There is growing concern from farmers, particularly those with land to the north of the park, that having too many beaver dams increases the risk of flooding on their fields. This concern is not unfounded, as we show dam capacity is high along the northern boundary of the park as this is where the lowlands physiography occurs. Given there is the potential for a large volume of water to be impounded near the northern park boundary, farmers are pressuring park managers to remove beaver dams from this area as they fear the kinds of destructive outburst floods created by beaver dam failure that Hillman^49^ described.

Current beaver dam density on streams in Riding Mountain National Park is only 22% of the capacity predicted by the BRAT model. It seems unlikely that the beaver population is still recovering from the fur trade as beaver cache counts in the park are relatively steady^50^. There are several other possible reasons why beaver build fewer dams than the model predicts can be build. Every model has limitations. Some of the parameters the BRAT model does not account for are wet and dry cycles, common in the boreal plain^18^, or the fine-scale variations in habitat features, like the presence of deep pools^51^, that are important in influencing the number of dams a beaver colony will build along a stream reach at any time. Some of these challenges may be overcome by calculating the probability of dam construction within a Bayesian framework, such as was recently carried out in Graham et al.^52^. Also, beaver in Riding Mountain National Park compete with ungulates (primarily moose and elk) for woody resources, which would limit their population and thus ability to build dams^53^. Further, the park is actively managing beaver in places near hiking trails, buildings and roads. While management practices include both killing beaver and dismantling dams (R. Baird, pers. comm.), these practices are occurring in localized areas only and so are unlikely to significantly impact the overall number of beaver dams in the park.

Importantly, we observed many beaver dams not on stream channels, especially in the hummocky physiography. These dams are occurring away from streams, similar to what is reported to occur in mountain environments^54^. This is not surprising as the boreal forest is known to consist of many drainage pathways that are not mapped as streams^9^. In particular, hummocky parts of the boreal landscape tend to have a disjointed drainage pattern and high density of open water bodies^55^. Beaver also readily inhabit ponds^20^ and groundwater seeps^56^ which are abundant in the boreal plain. Dams built in these environments are critical locations in boreal plains watersheds for surface water storage^39^, and they are also likely important places of groundwater recharge^10^. But, beaver dams not located within the stream network are not captured by the BRAT model. While these dams clearly have a different hydrological role in the landscape than those located on stream channels, they do contribute overall to beaver-mediated hydrological modification of the boreal forest and so improvements to the BRAT model are needed to make it a widely applicable tool.

The second implication of our results involves changes in forest composition that alter the park’s beaver dam capacity. Although we did not identify specific causes of forest change over the study period, the composition and structure of the boreal forest naturally changes over time. Boreal forests are disturbance-driven ecosystems with fire, for example, being an integral part of a healthy boreal ecosystem^57^. Riding Mountain National Park uses prescribed burning and also selective logging as part of their park management plan^58^. Fires can renew beaver food resources. However, aspen does not become abundant in the Canadian boreal forest until 10–15 years post fire disturbance^59^, and it might take several decades for beaver colonies to flourish post fire disturbance as population recovery lags vegetation recovery^24^. As well, beaver play a role in altering forest composition—they are well known to create and maintain vegetation conditions conducive to their reoccupation through flooding and selective foraging^60^. When beavers are trapped out of ecosystems, forests transition to conifer-dominated, which is not palatable to beaver^61^. Understanding how past forest change impacts beaver dam capacity over time is a crucial first step to understanding how beaver dam capacity could be impacted in the future, and is an area requiring further research.

The third implication of our work is that future changes in climate will impact the dam-building capacity of boreal beavers. Looking forward, the climate future of the boreal plains is expected to be quite different than it has been. A future warmer climate will alter the water cycle, including the precipitation to evaporation regime^18,23,25,62^. Already, this part of Canada’s boreal forest is noticeably wetter than it was a few decades ago^39^, which is causing nonlinear increases in streamflow^63^. The modelling results under scenarios of increased streamflow indicate we should expect a reduction in beaver dam capacity and thus in beaver-mediated water storage. The modelling results indicate that a doubling of the size of the 2-year flood will reduce beaver dam capacity in RMNP by 21%. The BRAT model classifies a stream reach as not capable of supporting beaver dams where stream power exceeds 2000 W/m; a threshold above which McFarlane et al.^34^ indicate beaver dams commonly fail. However, there is significant uncertainty around dam failure threshold. Some studies report that higher streamflow, especially during spring snowmelt, can lead to higher rates of beaver dam failure^64^ while other studies report low rates of dam failure during large floods^65,66^. Thus, the dam failure threshold probably varies widely based on the state of repair of a dam, the materials from which the dam was built and pond fullness at the onset of a rain event^11^.

Overall, the study results bring new insights into the ability of beaver to offset some of the predicted effects of climate change. Specifically, it is the dam modification of stream hydrology that has led to a growing reliance on beaver in climate change adaptation (and stream restoration) strategies^21^. However, there are numerous interacting processes regulating where and how many dams beaver built along stream networks^51^. Our model results suggest climatic changes to streamflow regimes and forest composition will cause a feedback whereby the capacity of stream networks to support beaver dams is diminished. Thus, our findings are inconsistent with the beaver population model predictions of Jarema et al.^29^ whereby climate changes will lead to substantial increases in beaver density within the interior of their range. However, a direct link between the beaver population and the number of dam built remains to be shown.

## Methods

### Study area

The study was carried out in Riding Mountain National Park, Canada, located at the southeastern limit of the boreal plain (Fig. 1). Riding Mountain National Park is 2976 km^2^ and is part of a larger biosphere reserve which encompasses the surrounding multi-use landscape. Riding Mountain National park has 2604 km of streams, distributed unevenly across its four physiographies: escarpment (721 km), lowlands (229 km), hummocky (208), and upland plateau (1446 km). The upland plateau comprises most of the park and is characterized by rolling hills with an uneven distribution of glacial till and thus consists of many poorly-drained wetlands^67^. The Manitoba escarpment is characterized by eroded shale bedrock dissected by deep ravines produced by streams draining the upland plateau. The lowlands district is located at the base of the escarpment and is dominated by eastern deciduous forests where flooding is common and fire disturbance is rare. The hummocky district occurs in the southern and western reaches of Riding Mountain National Park and is characterized by numerous wetlands created by melting blocks of ice left by the retreating glaciers. Forest cover in Riding Mountain National Park is boreal mixedwood. Key tree species include white spruce (*Picea glauca*), trembling aspen (*Populus tremuloides*), balsam poplar (*P. balsamifera*), paper birch (*Betula papyrifera*) and black spruce (*Picea mariana*)^68^. Dominant shrubs include beaked hazelnut (*Corylus cornuta*) and mountain maple (*Acer spicatum*) in well-drained sites, and alder (*Alnus* spp.) in moist sites^58^.

### Modelling beaver dam capacity

We modelled beaver dam capacity using the Beaver Restoration Assessment Tool (BRAT) model^34^, version PyBRAT 3.0.17, available at: https://github.com/Riverscapes/pyBRAT/releases/. BRAT is a GIS-based model that combines stream segment slope, beaver vegetation preferences and streamflow information to predict the upper limits of stream reaches to support beaver dams, and then projects these limits onto a stream network. The BRAT model was chosen over other beaver occupancy models as it relies on datasets that are less precise than those required for statistically-based models, and so can be used across large areas and in landscapes with different characteristics. The requisite inputs to the beaver dam capacity model include a drainage network layer, vegetation type raster data of historic and existing conditions, a digital elevation model (DEM) raster, and streamflow (baseflow and peak flow) information throughout the drainage network. Methods by Macfarlane et al.^34^ were followed using ESRI ArcMap 10.5 with minor customizations including the preprocessing of BRAT vegetation and stream power data to customize the tool to the boreal forest generally and Riding Mountain National Park specifically.

### Vegetation input

BRAT modelled output of dam capacity is driven by vegetation data and constrained by stream power and stream gradient. Historic and existing vegetation layers for a study site are required inputs to the BRAT model. BRAT was created to use the publicly available datasets in the United States. These are not necessarily available in Canada. So, we used previously classified landcover maps of Riding Mountain National Park from 1991, 2003, and 2016 provided by Riding Mountain National Park. The imagery analysis for 1991 was created using a 30 m resolution LANDSAT 5 image acquired in August of 1991. The vegetation layer from 2003 was created using LANDSAT 5 Thematic Mapper (TM) imagery at a 30 m resolution; the month of that imagery was acquired was not available. Imagery from 2016 was derived from Sentinel 2 satellite imagery from June and July 2016 at a 10 m resolution. Since the vegetation raster for 2016 was at a 10 m resolution, the aggregate tool in ESRI ArcMap 10.5 was used with a cell factor of ‘3’ and the aggregation type kept as the default (‘SUM’). The output was a reduced-resolution version of 30 m.

To tailor beaver vegetation selection based on vegetation common in the boreal forest, we modified Macfarlane et al.’s^34^ vegetation suitability classification. We added a “VEG_CODE” field in each of the three vegetation raster files by adding a field code to each layer’s attribute table within ESRI ArcMap. The ‘VEG_CODE” field allows for each landcover type to be customized to the beaver preference. A value of 0–4 was assigned to each vegetation classification type based on its suitability as dam building material, with 0 being ‘unsuitable’, 1 being ‘barely suitable’, 2 being ‘moderately suitable’, 3 being ‘suitable material and 4 being ‘preferred material’.

### Stream power input

The BRAT model uses stream power to determine the likelihood of a beaver dam persisting along a stream reach during low flow (Qlow) and 2-year flood (Q2) conditions^34^. There are no stream gauges within the park. Thus, in order to apply the BRAT model in RMNP, we chose the nearest Water Survey of Canada hydrometric station (gauge 05LJ045) located to the north of the park and used the daily and annual discharge record from it (1979–2015) in a regional flood frequency analysis. To calculate Qlow, 7Q10 was calculated using the R package ‘Flowscreen’ which used the lowest discharge reading and the subsequent measurement for 6 days for each hydrologic year and then calculated the mean discharge. Using the Qlow and Q2 discharge values for gauge 05LJ045, we edited the ‘iHyd’ python script which is a part of the BRAT toolbox in ESRI ArcMap 10.5 in IDLE. The ‘iHyd’ tool prepares the hydrologic inputs to BRAT by taking the equations created for Qlow and Q2 and calculates the values using the drainage-discharge relationship. The tool adds on two additional fields in the attribute table called ‘iHyd_SPLow’ and ‘iHyd_SP2’. Stream power was subsequently calculated as:

1 $$\Omega = \rho \cdot {\text{g}} \cdot {\text{Q}} \cdot {\text{S}}$$

where Ω is the total stream power (W/m), ρ is water density (1000 kg/m^3^), g is gravitational acceleration (9.8 m/s^2^), Q is discharge (m^3^/s) and S is steam bed slope, which was calculated using the DEM to estimate the highest and lowest points along each 300 m stream segment.

The BRAT calculates maximum number of beaver dams each stream segment can support, based on the stream power (Qlow and Q2), vegetation, and stream segment slope. Using the calculated maximum number of beaver dams, each stream segment is subsequently classified into the following categories; ‘Pervasive—16 to 40 dams’ (blue), ‘Frequent—5 to 15 dams’ (green), ‘Occasional—1 to 4 dams’ (yellow), ‘Rare—0 to 1 dams’ (orange) and ‘None’ (red). The model also outputs a polyline shapefile of the original drainage network with all the above attribute data added to it and symbology layers to display classified the network in ESRI ArcMap.

### Water storage

Beaver ponds in the park were surveyed to determine their water storage. As Riding Mountain National Park is large, and automated beaver pond detection techniques do not yet exist, we manually identified and delineated ponds present on the most recent, high-resolution aerial imagery (2004; 0.625 m resolution; “SI1”). The pond inventory was done within the long-term set of 30 randomly distributed survey blocks, each 24 km^2^, that the Canadian Wildlife Service established in 1973 to monitor beaver caches. Survey blocks were divided into six equal quadrants (~ 4 km^2^) and one quadrant per survey block was randomly selected for analysis. The total number and surface area (m^2^) of beaver ponds in each selected quadrant were calculated using the ‘calculate geometry’ feature in ArcMap 10.5 (see “SI1”). We used the bootstrap technique (10,000 iterations), resampled from the observations, to estimate the beaver pond size distribution for the park. Pond sizes fit a negative exponential distribution—median pond surface area (x) was 1419 m^2^, and the 95% confidence interval was [1157, 1704 m^2^] (see “SI2”). Water storage volume (V) was calculated using Karran et al.^35^

2 $$V = 0.0955x^{1.1562}$$

for a median-sized beaver pond and its 95% confidence interval. To estimate beaver pond water storage capacity for the entire park, we multiplied the volume of water stored in the median sized pond (421 m^3^; 95% confidence interval [333, 520 m^3^]) by the BRAT estimate of beaver dam capacity (see “SI2”).

### Changing vegetation composition

To determine whether the vegetation changes over time were real or an artifact of vegetation classification differences in the different years, LANDSAT images from each year were analyzed in ArcMap 10.5 using the time series change detection method Landsat-based detection of Trends in Disturbance and Recovery (‘Landtrendr’) outlined in Kennedy et al.^68^. The normalized burn ratio (NBR) spectral index was used to determine the disturbance magnitude or cover percentage lost due to disturbance. This approach examines each pixel from year to year to see how it changes over time and highlights it if a substantial change is detected. Additional analysis was undertaken on the highlighted pixels to determine what type of cover change occurred, either a full cover change type or a shift in the spectral index value—indicating cover degradation. Results were compiled into a database to calculate the total percentage of changes. Additionally, results were projected in ESRI ArcMap as shapefiles, to highlight areas of change.

### Stream flow changes

Published research predicts significant future stream flow changes in the boreal plains ecozone^19,63^. To evaluate how climate-induced changes to the streamflow hydrological regime might impact beaver dam capacity, we manipulated the discharge (Qlow and Q2) values in BRAT. Scenarios run included a Qlow increase of 25% and a decrease of 25%. Also, Q2 was increased by 10%, 25% and 100% to simulate the effects of larger 2-year floods. To run the scenarios, we edited the ‘iHyd’ python script in IDLE to include the new discharge rates and ran the BRAT model for each scenario. There were no differences in total dam capacity with the Qlow scenarios as all scenarios resulted in stream powers between 0 and 175 W/m, which BRAT designates as ‘can build dam’. Meaning, decreasing Qlow does not impact the model results, and as a result, BRAT does not recognize any stream reaches being unable to support beaver dams. Thus, reported are only scenarios where Q2 was manipulated.

## Supplementary information

Supplementary file1

## Data Availability

The supplemental information and all non-publicly available data analyzed during this study are available in the following repository: https://github.com/Ecohydrology-westbrook/Stoll-Westbrook-Supplementary-Info.
