# Supplementary material for: Beaver dam capacity of Canada’s boreal plain in response to environmental change
Source: Sci Rep. 2020 Oct 8;10:16800. doi: 10.1038/s41598-020-73095-z (PMC7546727; doi:10.1038/s41598-020-73095-z)
Supplement: Supplementary file 1 — Supplementary file1 [file 41598_2020_73095_MOESM1_ESM.pdf]

Beaver dam capacity of Canada's boreal plain in response to environmental change

Supplementary Information

Nichole-Lynn Stoll[1], Cherie J. Westbrook[1]*¶

[1]Department of Geography and Planning, University of Saskatchewan, Saskatoon, SK, Canada S7N 5C8

*Correspondence to: cherie.westbrook@usask.ca

The datasets generated and/or analysed during the current study are available in the following repository:

https://github.com/Ecohydrology-westbrook/Stoll-Westbrook-Supplementary-Info
